# Pregnancy outcome after use of cranberry in pregnancy – the Norwegian mother and child cohort study

**DOI:** 10.1186/1472-6882-13-345

**Published:** 2013-12-07

**Authors:** Kristine Heitmann, Hedvig Nordeng, Lone Holst

**Affiliations:** 1 Department of Global Public Health and Primary Care and Centre for Pharmacy, University of Bergen, Norway, P.O. Box 7804, Bergen 5020, Norway; 2 Department of Pharmacy, School of Pharmacy, University of Oslo, P.O. Box 1068 Blindern, Oslo 0316, Norway; 3 Division of Mental Health, Norwegian Institute of Public Heath, P.O. Box 4404 Nydalen, Oslo 0403, Norway

**Keywords:** Pregnancy, Cranberry, Urinary tract infection, Herbal medicine, Antibiotics, Congenital malformations, Pregnancy outcome

## Abstract

**Background:**

Cranberry is one of the most commonly used herbs during pregnancy. The herb has been used traditionally against urinary tract infections. No studies are found that specifically address the risk of malformations after use of cranberry during pregnancy. The aim of the study was to investigate the safety of cranberry use during pregnancy, including any effects on congenital malformations and selected pregnancy outcomes.

**Methods:**

The study is based on data from The Norwegian Mother and Child Cohort Study including more than 100,000 pregnancies from 1999 to 2008. Information on use of cranberry and socio-demographic factors was retrieved from three self-administered questionnaires completed by the women in pregnancy weeks 17 and 30, and 6 months after birth. Information on pregnancy outcomes was retrieved from the Medical Birth Registry of Norway.

**Results:**

Among the 68,522 women in the study, 919 (1.3%) women had used cranberry while pregnant. We did not detect any increased risk of congenital malformations after use of cranberry. Furthermore, the use of cranberry was also not associated with increased risk for stillbirth/neonatal death, low birth weight, small for gestational age, preterm birth, low Apgar score (<7), neonatal infections or maternal vaginal bleeding in early pregnancy. Although an association was found between use of cranberry in late pregnancy and vaginal bleeding after pregnancy week 17, further sub-analyses of more severe bleeding outcomes did not support a significant risk.

**Conclusions:**

The findings of this study, revealing no increased risk of malformations nor any of the following pregnancy outcomes; stillbirth/neonatal death, preterm delivery, low birth weight, small for gestational age, low Apgar score and neonatal infections are reassuring. However, maternal vaginal bleeding should be investigated further before any firm conclusion can be drawn. Treatment guidelines on asymptomatic bacteriuria in pregnancy recommend antimicrobial therapy as the first line treatment. According to our data and the outcomes studied, cranberry does not appear to be a harmful adjunctive self-treatment.

## Background

Cranberry (*Vaccinium macrocarpon* Aiton), also known as American Cranberry, is a fruit native to North America, and has been used traditionally against urinary tract infections (UTIs) [1]. It is included in the United States Pharmacopeia as Cranberry Liquid Preparation [2], and is usually administered as cranberry juice cocktail or as capsules. Cranberry is one of the most commonly used herbs during pregnancy, and five studies report prevalence rates of over 5% [3-7].

UTIs are the most common bacterial infections in pregnancy and may be classified as asymptomatic or symptomatic [8]. The incidence of asymptomatic bacteriuria (ASB) in pregnancy has been reported to be 2–14%, similar to the incidence in non-pregnant women [9]. However, pregnant women have an increased risk of progression of ASB into pyelonephritis compared to non-pregnant women, 20–40% vs. 1–2%, respectively [9]. The risk of ascending infection is increased because of dilated ureters caused by the mechanical effects of the enlarged uterus and increased levels of progesterone [10]. Symptomatic urinary tract infections are classified as lower tract (cystitis), and upper tract infections (pyelonephritis), which occur in 1–4% and 0.5–2% of all pregnancies, respectively [8,9].

The consequences of UTI on pregnancy outcomes are well documented. Bacteriuria has been associated with an increased risk of preterm birth, intrauterine growth restriction and low birth weight in several studies [11-14]. Pyelonephritis is the second most common, and a serious, medical problem in pregnancy [10]. It has been associated with different perinatal complications and adverse pregnancy outcomes including bacteraemia, septic shock, respiratory insufficiency, anaemia, renal disease, hypertension, preterm delivery and low birth weight [8,12,13,15,16]. Use of antibiotics against ASB has been shown to reduce the risk of low birth weight [11,17]. Thus, guidelines on UTI in pregnancy clearly state that UTI should be treated with antibiotics [18,19].

Cranberries’ mechanism of action has not been fully elucidated, though it is thought to be attributed to two components, proanthocyanidins and fructose [20]. These two components have been shown to inhibit adherence of bacteria to the cells lining the wall of the bladder [21-23], but do not seem to have the ability to release bacteria already adhered to those cells [24]. According to a Cochrane review, there is no scientific evidence to support the use of cranberry for treating UTIs [25]. Until recently, there was some evidence supporting the use of cranberry in prevention of recurrent UTIs, especially in women [26], but the recently updated Cochrane review states that, currently, there is insufficient evidence to recommend cranberry for prevention of recurrent UTIs [27]. However, pooled results from two studies comparing a cranberry product with antibiotics showed no significant difference between groups in terms of risk of recurrent UTI [27].

The widespread use in pregnancy is in contrast to the sparse body of evidence on both safety and effectiveness of cranberry use during pregnancy against UTIs. One study investigating the efficacy of cranberry for the prevention of ASB in pregnancy is found [28]. This was a three-arm study comparing a single daily dose (240 ml) or two/three daily doses (480 ml to 720 ml) of cranberry juice cocktail with a placebo beverage. Though not significant, the authors found a trend of fewer UTIs in the group ingesting two/three doses of cranberry juice cocktail compared to the group ingesting a single daily dose and the placebo group (6.9% vs. 11.1% and 10.4%, respectively). This study did not detect any differences between the groups with regards to obstetrical or neonatal outcomes such as preterm delivery, route of delivery, birth weight, Apgar score or admission to neonatal intensive care unit. No studies are found that specifically address the risk of malformations after use of cranberry during pregnancy. Though textbooks refer to the long history of safe use and no known negative reports regarding the use of cranberry during pregnancy, it is also stated that there is a need for evidence based documentation [29-31]. No case reports of negative pregnancy outcome are discovered. However, since cranberry probably is ineffective in treating UTIs in pregnancy, it is possible that using cranberry as replacement for antibiotics will have negative consequences on pregnancy outcome due to the untreated underlying infection.

Previous literature indicates a possible interaction between cranberry and warfarin, and different mechanisms have been postulated [20,32,33]. Although elevations of the international normalized ratio (INR) after consumption of cranberry have been described in a number of case reports, results from clinical trials have been conflicting [32,34]. It is unknown whether ingestion of cranberry may increase the risk of bleeding in other vulnerable patient groups, e.g. pregnant women.

The primary aim for this study was to investigate whether use of cranberry during pregnancy was associated with an increased risk of malformations. The secondary aim was to examine whether use of cranberry during pregnancy was associated with selected pregnancy outcomes such as stillbirth/neonatal death, low birth weight, preterm birth, low Apgar score, neonatal infections and maternal vaginal bleeding.

## Methods

The data used in this study was provided by the Norwegian Mother and Child Cohort Study (MoBa) and the records from the Medical Birth Registry of Norway (MBRN). Access to MoBa was granted by the MoBa steering committee at the National Institute of Public Health according to regulations for MoBa-substudy projects. MoBa is a population-based prospective cohort study initiated by and maintained at the Norwegian Institute of Public Health including more than 100,000 pregnancies from 1999 to 2008 [35]. Pregnant women received a postal invitation including an informed consent form and the first questionnaire together with an appointment for a routine ultrasound examination during pregnancy week 17–18 [36]. The recruitment originated in the county of Hordaland and expanded gradually to become nationwide in 2005, with 50 of 52 hospitals in Norway participating. The participation rate was 43.8% according to an assessment of MoBa in June 2009 [36]. Three self-administered questionnaires from MoBa were used to retrieve information [37-39]. The first and second questionnaires were completed during pregnancy weeks 13–17 and 30 [37,38], respectively, and the third was completed when the child was 6 months old [39]. The questionnaires provide information on socio-demographic characteristics, outcomes of previous pregnancies, medical history, maternal health, lifestyle habits, drug exposure, and other exposures, e.g. use of herbal products. Among respondents who participated, the response rate was 95% for the first questionnaire, 92% for the second questionnaire, and 87% for the third questionnaire [36].

The cohort database is linked to the MBRN via the woman’s 11 digit personal identification number, which is assigned to individuals registered in the National Population Register as residents of Norway. Since the establishment of MBRN in 1967 it has been compulsory to notify the register of every birth or late abortion, including live birth, stillbirth and induced abortions after gestational week 12 (after week 16 up to 2002), occurring in Norway [40,41]. The registry contains information on pregnancy, delivery, and the health of the neonate [41].

MoBa was approved by the Regional Committee for Ethics in Medical Research, Region South, and the Norwegian Data Inspectorate.

### Study population

The current study uses version 4 of the MoBa quality assured data files made available for research. This file includes 72,934 women who delivered between 1999 and 2006. To be included in the current study, the women had to have a record in MBRN and to have answered the first questionnaire (n = 69,930). Women who gave birth to multiples (n = 1,291) or who gave birth to infants with chromosomal malformations (n = 121) were excluded. This resulted in a study population of 68,522 pregnant women and their newborn infants, corresponding to 94.0% of the original data file. Among the included women, 92.5% had answered the second questionnaire and 87.3% had answered the third questionnaire.

### Exposure variable

Information on cranberry use was retrieved from the three MoBa questionnaires [37-39]. In each questionnaire, it was asked if the women had experienced specified complaints, including UTI. The women could specify several products used and exposure windows for each separately, when the complaint was experienced and when the products were used; in the first questionnaire: 6 months before pregnancy, gestational weeks 0–4, 5–8, 9–12 and 13+ (until completion of the first questionnaire); in the second questionnaire: gestational weeks 13–16, 17–20, 21–24, 25–28, 29+ (until completion of the second questionnaire); and in the third questionnaire: last part of pregnancy, 0–3 months after birth and 4–6 months after birth. In addition, the questionnaires included a question about use of all vitamins and dietary supplements, including alternative/herbal remedies, in which the women were asked to give complete names of the products. In this question, it was not possible to specify either timing of use or indication. All medicine text fields and text fields for dietary supplements/herbal remedies in the three questionnaires were systematically reviewed for herbal products by the research team (see also acknowledgement). Whenever a herbal product was identified, the herbal ingredient(s) was systematically coded according to a pre-determined herbal classification list. This classification system had been developed by the research team and included the common name of the herb and a seven character specific code as a means to standardize the coding in the questionnaire database. Cranberry was defined as any herbal coded with the assigned herbal code 59.

Exposure was classified as use of cranberry during pregnancy (total), use of cranberry during early pregnancy (before pregnancy week 17) and use of cranberry during late pregnancy (at and after pregnancy week 17). Sub-analyses stratifying on timing of use and co-medication with antibiotics were performed.

### Outcome variables

Information on outcome variables was abstracted from the MBRN. Diagnoses were based on the International Classification of Diseases, 10^th^ Revision (ICD-10) [42]. Malformations were classified as all malformations, major malformations and cardiovascular malformations. Malformations (all malformations) were defined as any birth defect registered in the MBRN [43]. Malformations were classified as major by the MBRN using the International Clearinghouse of Birth Defects definition [43]. Cardiovascular malformations were defined as all malformations classified with ICD-10 code Q20-26 [43].

Other outcome variables included in the study were stillbirth/neonatal death, low birth weight (<2500 g), small for gestational age (SGA) (under the 10^th^ percentile for the gestational age), preterm birth (<37 weeks gestational age), Apgar score <7 at 5 minutes after birth and neonatal infections. Stillbirth was defined as the death of a foetus with a gestational age of ≥22 weeks or with a birth weight of ≥425 g. Neonatal death was defined as a live born infant that died within the first 28 days after birth. Neonatal infections were defined as all diagnoses classified with ICD-10 P35-39. The outcomes were not mutually exclusive.

The MoBa study provided information on the different variables related to maternal vaginal bleeding: vaginal bleeding before pregnancy week 17, vaginal bleeding at week 17 and after, hospitalization due to vaginal bleeding before week 17, hospitalization due to vaginal bleeding at week 17 and after, vaginal bleeding more than spotting after week 17.

### Other variables

The following potential confounders were explored in relation to cranberry use and the different outcome variables: maternal age (≤24 years,25–29 years, 30–34 years, ≥35 years); parity [0 previous live births, ≥1 previous live birth(s)], education (primary, secondary, tertiary-short, tertiary-long); marital status (married or cohabitating, other); pre-pregnancy body mass index (BMI) underweight (<18.5 kg/m^2^), normal weight (18.5 – 24.99 kg/m^2^), overweight (25.0 – 29.99 kg/m^2^), obese (≥ 30 kg/m^2^); physical activity (never, less than once a week, 1–2 times weekly, 3 or more times weekly); maternal mother tongue (Norwegian, other); smoking at the end of pregnancy (no, sometimes, daily); any folic acid use (no; yes, before or during; yes, before and during), and year of delivery (1999–2002, 2003–2006).

Maternal UTI was classified as UTI during early pregnancy (before week 17), UTI during late pregnancy (at week 17 and after), and UTI during pregnancy at any time point (total). In addition, sick leave, previous miscarriages or stillbirths, use of antibiotics against UTI and maternal vaginal infections including vaginal thrush, vaginal catarrh/ unusual discharge and/or chlamydia, were variables that were included. These variables were dichotomized yes/no.

### Statistical analyses

Pearson’s chi-square test was used to explore potential associations between maternal characteristics listed in Table 1 and cranberry use. In cases where the expected value in any of the cells of a contingency table was below 5, Fisher’s exact test was applied. P-values of <0.05 were considered significant. To estimate the risk of malformations and selected pregnancy outcomes, crude and adjusted odds ratios (ORs) were obtained by performing univariate and multivariate generalized estimating equations. ORs are presented with 95% confidence intervals (CIs). The generalized estimating equation with the binary logistic model was used to correct for possible correlations between pregnancies if the women had participated with more than one pregnancy. In total, 6,972 (10.2%) women had participated with more than one pregnancy. Of note, we also performed univariate and multivariate logistic regression analyses and compared the effect estimates obtained to those obtained by generalized estimating equations. However, the two effect estimates differed only slightly, hence the ORs and CIs calculated by using generalized estimating equations are presented in the text and tables. The variables listed in Table 1 were considered potential confounders. Statistically or clinically significant variables were explored for each pregnancy outcome. The selection of variables to be included in the potential confounder sets was based on theoretically potential influences, as well as the results from exploratory data analysis. Possible high inter-correlations among the independent variables were checked for, using multiple regression analysis and ensuring that the tolerance values for collinearity statistics were adequate (>0.1).

**Table 1:** **Characteristics of women according to cranberry use, n = 68,522**^**a**^

	**Total**	**No cranberry use during pregnancy**	**Use of cranberry during pregnancy**		**No exposure to cranberry and UTI**	**Exposed to cranberry, UTI and antibiotics against UTI during pregnancy**	**Exposed to cranberry, UTI and no antibiotics against UTI during pregnancy**	
	***No. (%)***	***No. (%)***	***No. (%)***		***N (%)***	***N (%)***	***N (%)***	
	**68,522 (100.0)**	**67,603 (98.7)**	**919 (1.3)**	**P-value**^**b**^	**60,846 (88.8)**	**254 (0.4)**	**300 (0.4)**	**P-value**^**b**^
**Age (years)**								
≤ 24	8,034 (11.7)	7,930 (11.7)	104 (11.3)	0.621	6,893 (11.3)	28 (11.0)	32 (10.7)	0.952
25–29	23,050 (33.6)	22,725 (33.6)	325 (35.4)		20,442 (33.6)	88 (34.6)	106 (35.3)	
30–34	26,157 (38.2)	25,822 (38.2)	335 (36.5)		23,454 (38.5)	92 (36.2)	110 (36.7)	
≥ 35	11,281 (16.5)	11,126 (16.5)	155 (16.9)		10,057 (16.5)	46 (18.1)	52 (17.3)	
**Parity**								
0 previous live births	29,778 (43.5)	29,272 (43.3)	506 (55.1)	**<0.001**	26,090 (42.9)	152 (59.8)	147 (49.0)	**<0.001**
≥1	38,738 (56.5)	38,325 (56.7)	413 (44.9)		34,750 (57.1)	102 (40.2)	153 (51.0)	
**Education**^**c**^								
Primary	6,123 (8.9)	6,060 (9.0)	63 (6.9)	**<0.001**	5,287 (8.7)	14 (5.5)	26 (8.7)	**0.032**
Secondary	20,519 (29.9)	20,261 (30.0)	258 (28.1)		18,069 (29.7)	64 (25.2)	97 (32.3)	
Tertiary-short	27,204 (39.7)	26,777 (39.6)	427 (46.5)		24,264 (39.9)	128 (50.4)	120 (40.0)	
Tertiary-long	13,112 (19.1)	12,956 (19.2)	156 (17.0)		11,817 (19.4)	45 (17.7)	50 (16.7)	
**Marital status**								
Married/cohabitating	65,765 (96.0)	64,878 (96.0)	887 (96.5)	0.320	58,536 (96.2)	246 (96.7)	288 (96.0)	0.834
Other	2,427 (3.5)	2,400 (3.6)	27 (2.9)		2,017 (3.3)	7 (2.8)	11 (3.7)	
**Pre-pregnancy BMI**^**d**^								
Underweight	2,055 (3.0)	2,022 (3.0)	33 (3.6)	0.195	1,799 (3.0)	7 (2.8)	15 (5.0)	0.218
Normal weight	43,058 (62.8)	42,446 (62.8)	612 (66.6)		38,377 (63.1)	167 (65.7)	178 (59.3)	
Overweight	14,736 (21.5)	14,558 (21.5)	178 (19.4)		13,052 (21.5)	50 (19.7)	67 (22.3)	
Obese	6,538 (9.5)	6,452 (9.5)	86 (9.4)		5,701 (9.4)	26 (10.2)	37 (12.3)	
**Physical activity**								
Never	10,293 (15.0)	10,164 (15.0)	129 (14.0)	0.417	9,080 (14.9)	31 (12.2)	45 (15.0)	0.538
Less than once a week	16,218 (23.7)	16,000 (23.7)	218 (23.7)		14,423 (23.7)	57 (22.4)	65 (21.7)	
1-2 times weekly	25,747 (37.6)	25,369 (37.5)	378 (41.1)		22,931 (37.7)	111 (43.7)	124 (41.3)	
3 times or more weekly	11,121 (7.5)	10,969 (16.2)	152 (16.5)		9,871 (16.2)	42 (16.5)	50 (16.7)	
Missing	5,143 (7.5)	5,101 (7.5)	42 (4.6)		4,541 (7.5)	13 (5.1)	16 (5.3)	
**Maternal mother tongue**								
Norwegian	64,812 (94.6)	63,929 (94.6)	883 (96.1)	**0.043**	57,511 (94.5)	248 (97.6)	287 (95.7)	0.064
Other	3,710 (5.4)	3,674 (5.4)	36 (3.9)		3,335 (5.5)	6 (2.4)	13 (4.3)	
**Smoking at the end of pregnancy**								
No	53,198 (77.6)	52,468 (77.6)	730 (79.4)	**0.024**	47,283 (77.7)	202 (79.5)	236 (78.7)	0.623
Sometimes	472 (0.7)	462 (0.7)	10 (1.1)		409 (0.7)	0 (0.0)	3 (1.0)	
Daily	3,524 (5.1)	3,492 (5.2)	32 (3.5)		3041 (5.0)	10 (3.9)	14 (4.7)	
Missing	11,328 (16.5)	11,181 (16.5)	147 (16.0)		10,113 (16.6)	42 (16.5)	47 (15.7)	
**Any folic acid use**^**e**^								
No	32,098 (46.8)	31,715 (46.9)	383 (41.7)	**0.007**	28,356 (46.6)	108 (42.5)	123 (41.0)	**0.044**
Yes, before or during	21,735 (31.7)	21,417 (31.7)	318 (34.6)		19,294 (31.7)	96 (37.8)	97 (32.3)	
Yes, before and during	14,689 (21.4)	14,471 (21.4)	218 (23.7)		13,196 (21.7)	50 (19.7)	80 (26.7)	
**Year of delivery**								
1999-2002	13,640 (19.9)	13,482 (19.9)	158 (17.2)	**0.038**	11,985 (19.7)	43 (16.9)	69 (23.0)	0.192
2003-2006	54,882 (80.1)	54,121 (80.1)	761 (82.8)		48,861 (80.3)	211 (83.1)	231 (77.0)	
**Sick leave**								
Yes	43,300 (63.2)	42,693 (63.2)	607 (66.1)	0.070	38,147 (62.7)	172 (67.7)	198 (66.0)	0.128
**Previous miscarriages/stillbirths**								
Yes	14,975 (21.9)	14,786 (21.9)	189 (20.6)	0.341	13,279 (21.8)	49 (19.3)	57 (19.0)	0.311
**Vaginal infections**^**f**^								
Yes	21,924 (32.0)	21,514 (31.8)	410 (44.6)	**<0.001**	18,642 (30.6)	125 (49.2)	145 (48.3)	**<0.001**
**Urinary tract infection (UTI)**								
UTI during early pregnancy^g^	4,624 (6.7)	4,222 (6.2)	402 (43.7)	**<0.001**	-	-	-	
UTI during late pregnancy^h^	3,766 (5.5)	3,462 (5.1)	304 (33.1)	**<0.001**	-	-	-	
UTI during pregnancy at any time point	7,311 (10.7)	6,757 (10.0)	554 (60.3)	**<0.001**	-	-	-	

^a^Numbers may not add up to 68,522 due to missing values.

^b^P-values obtained by Pearson’s chi-square test or Fischer’s exact test when less than 5 in one or more of the cells. Significant findings are in bold.

^c^Primary: <10 years of education (the Norwegian compulsory primary + secondary school), secondary: 10–12 years (high school/upper secondary or vocational school), tertiary-short: college education, tertiary-long: university education.

^d^Body mass index (BMI) is the weight in kilograms divided by the square of the height in meters: underweight: <18.5 kg/m^2^, normal weight: 18.5–24.9 kg/m^2^; overweight: 25.0–29.9 kg/m^2^, obese ≥30 kg/m^2^.

^e^Folic acid use is reported from the 4 weeks prior to pregnancy to 3 months of gestation.

^f^Including vaginal thrush, vaginal catarrh/unusual discharge and/or chlamydia.

^g^Early pregnancy is defined as before pregnancy week 17.

^h^Late pregnancy is defined as during or after pregnancy week 17.

We also performed generalized estimating equations analyses stratifying on antibiotic treatment to further investigate the risk of the selected adverse pregnancy outcomes among the women who had used cranberry and who reported to have had at least one episode of UTI during pregnancy. The confounder sets used when estimating the risks for the adverse pregnancy outcomes were identical to the previously described, with the exclusion of UTI.

Given the 919 cases of exposures to cranberry during pregnancy, and the 566 exposures to cranberry during early pregnancy, the study had ≥80% statistical power to rule out a doubling or more of the risk of the following outcomes: malformations overall, serious malformations, low birth weight, low Apgar score, preterm birth and neonatal infections, and a tripling or more of the risk of the following outcomes: cardiac malformations and stillbirth/neonatal death.

The Statistical Package for Social Sciences version 20.0 (IBM SPSS Statistics 20) for Windows (SPSS, Chicago, IL, USA) was used to perform all the statistical analyses.

## Results

A total of 68,522 pregnancies were included in the study, of which 68,198 (99.5%) resulted in a live birth, 219 (0.3%) in a stillbirth, 104 (0.2%) in a neonatal death, and 24 (0.0004%) in an induced abortion after week 12. The overall rate of malformations was 4.7%. The mean birth weight was 3605 gram (standard deviation (SD) 590 g) and the median gestational age was 40 weeks among the live born neonates.

In total, 919 (1.3%) women reported use of cranberry during pregnancy. Among the women who had used cranberry, 121 (13.2%) women had used cranberry during the first trimester, and 566 (61.6%) women reported use during early pregnancy (in the first questionnaire). The women who had used cranberry were more likely to be primiparous, to have a college education (tertiary education-short), to have Norwegian as their maternal mother tongue and to have given birth during the time period 2003–2006, compared to the women who did not use cranberry (Table 1). In addition, women who had used cranberry were less likely to be daily smokers and non-users of folic acid.

The women who had used cranberry were more likely to have experienced UTI during pregnancy compared to women who did not use cranberry. In addition, a significantly higher proportion of the women who had used cranberry had experienced vaginal infections including vaginal thrush, vaginal catarrh/unusual discharge and chlamydia.

Among the women who had used cranberry, 554 (60.3% of 919) had experienced UTI at any time point during pregnancy. In total, UTI was experienced by 7,311 (10.7% of 68,522) of the women, of which 1,557 (21.3% of 7,311) women experienced more than one episode of UTI. Among the women who experienced UTI during pregnancy, 4,085 (55.9% of 7,311) reported using antibiotics against UTI compared to the 554 (7.6% of 7,311) women who reported use of cranberry. Furthermore, 300 women reported using cranberry and experiencing a UTI during pregnancy without the concomitant use of antibiotics.

Use of cranberry during early pregnancy did not increase the risk of vaginal bleeding before pregnancy week 17 (21.7% for users vs. 19.3% for non-users, p = 0.15). Additionally, use of cranberry during the first trimester was not associated with hospitalization due to vaginal bleeding (0.00% vs. 0.20%, p = 0.65). There was a higher percentage of bleeding after week 17 following ingestion of cranberry during the second and/or third trimester (9.7% vs. 5.8%, p < 0.001). However, no associations were found between use of cranberry during the second and/or third trimester and more severe bleeding outcomes such as vaginal bleeding more than spotting after pregnancy week 17 (3.1% vs. 2.1%, p = 0.08), or hospitalization due to vaginal bleeding after pregnancy week 12 (0.8% vs. 0.7%, p = 0.60). Furthermore, when adjusting for maternal age, parity, pre-pregnancy BMI, maternal smoking, maternal folic acid use, UTI, previous miscarriages or stillbirths, and physical activity, no associations were found between use of cranberry during the second and/or third trimester and bleeding after week 17 (adjusted OR 1.3, 95% CI 1.0–1.8, p =0.08) or vaginal bleeding more than spotting after week 17 (adjusted OR 1.2, 95% CI 0.8–2.1, p = 0.40).

No increased risk of overall malformations or major malformations was found after exposure to cranberry during early pregnancy (Table 2). There were no cases of cardiac malformations among the women who used cranberry during early pregnancy. Among the women who had used cranberry during the first trimester only, there were 11 (9.1% of 121) cases of malformations in total, of which five (4.1% of 121) were classified as major. However, sub-analyses with first trimester exposures only revealed no increased risk of overall malformations (adjusted OR 1.6, 95% CI 0.8–3.2) or major malformations (adjusted OR 1.3, 95% CI 0.5–3.6). The 11 cases of infants born with malformations after exposure to cranberry in the first trimester were scrutinized (data not shown). No specific pattern of malformations was revealed. The five cases of serious malformations were two cases of hypospadias, two cases of macrocephaly and one case of congenital deformity of the sternocleidomastoid muscle. The other cases which were not classified as serious malformations were four cases of congenital dislocation and other deformities of the hip (3 cases) and feet (1 case), one case of ankyloglossia and one case of undescended testicle.

**Table 2:** Association between malformations and exposure to cranberry during early pregnancy, n = 68,522*

		**No exposure to cranberry (n = 67,603)**			**Exposed to cranberry during early pregnancy**^**a **^**(n = 566)**		
**Outcome**	**Total (%)**	**No. (%)**	**Crude OR**	**Adjusted OR**	**No. (%)**	**Crude OR (95% CI)**	**Adjusted OR (95% CI)**
**Malformations, all**	3,201 (4.7)	3,145 (4.7)	Ref.	Ref.	33 (5.8)	1.3 (0.9–1.8)	1.1 (0.8–1.7)
**Major malformations**	1,777 (2.6)	1,753 (2.6)	Ref.	Ref.	11 (1.9)	0.7 (0.4–1.4)	0.7 (0.4–1.3)

*Abbreviations*: *CI* confidence interval, *OR* odds ratio, *Ref.* reference category.

*Odds ratios (ORs) resulting from Generalized estimating equations analyses are presented in the table.

^a^Early pregnancy is defined as before pregnancy week 17.

Malformations were defined according to the definitions of the MBRN and International Clearinghouse for Birth Defects.

Adjusted for maternal age, parity, pre-pregnancy BMI, level of education, maternal smoking, folic acid use, UTI, previous miscarriages or stillbirths, maternal mother tongue and year of delivery.

Use of cranberry during pregnancy did not seem to increase the risk of any of the selected negative pregnancy outcomes: stillbirth/neonatal death, low birth weight, small for gestational age, preterm birth, low Apgar score (<7) or neonatal infections (Table 3). Sub-analyses stratifying on timing of use were performed. The obtained adjusted ORs did not reveal any significant associations with the selected pregnancy outcomes by isolating cranberry exposure in early or late pregnancy (data not shown).

**Table 3:** Pregnancy outcome according to cranberry exposure, n = 68,522*

		**No exposure to cranberry (n = 67,603)**			**Exposed to cranberry during pregnancy (n = 919)**		
**Outcome**	**Total (%)**	**No. (%)**	**Crude OR**	**Adjusted OR**	**No. (%)**	**Crude OR (95% CI)**	**Adjusted OR (95% CI)**
**Stillbirth/neonatal death**^**a**^	323 (0.5)	319 (0.5)	Ref.	Ref.	4 (0.4)	0.9 (0.3–2.5)	1.1 (0.4–2.8)^g^
**Preterm birth**^**b**^	3,535 (5.2)	3,497 (5.2)	Ref.	Ref.	38 (4.1)	1.0 (0.8–1.4)	1.0 (0.8–1.4)^h^
**Low birth weight**^**c**^	2,182 (3.2)	2,160 (3.2)	Ref.	Ref.	22 (2.4)	0.7 (0.5–1.1)	0.7 (0.4–1.0)^g^
**Small for gestational age**^**d**^	4,281 (6.2)	4,226 (6.3)	Ref.	Ref.	55 (6.0)	1.0 (0.7–1.3)	0.8 (0.6–1.1)^i^
**Low Apgar score**^**e**^	898 (1.3)	887 (1.3)	Ref.	Ref.	11 (1.2)	1.2 (0.7–2.0)	1.0 (0.6–1.8)^g^
**Neonatal infections**^**f**^	870 (1.3)	856 (1.3)	Ref.	Ref.	14 (1.5)	1.2 (0.7–2.1)	1.1 (0.6–1.9)^j^

*Abbreviations: CI* confidence interval, *OR* odds ratio, *Ref*. reference category.

*Odds ratios (ORs) resulting from Generalized estimating equations analyses are presented in the table.

The outcomes were not mutually exclusive.

^a^Includes infants who were stillborn with a gestational age of ≥22 weeks or birth weight of ≥425 g, or who died during the first 28 days of life.

^b^Includes infants born at a gestational age of <37 weeks, which WHO defines as preterm.

^c^Includes infants with a birth weight of <2500 g, which WHO defines as low birth weight.

^d^Includes infants with a birth weight below the 10^th^ percentile at the attained gestational age.

^e^Includes infants who had an Apgar score of < 7 at 5 min after birth.

^f^Includes infants who were diagnosed with ICD-10 P35-39.

^g^Adjusted for maternal age, parity, pre-pregnancy BMI, folic acid use, smoking, UTI, education, previous miscarriages/stillbirths, length of gestation, maternal mother tongue and year of delivery.

^h^Adjusted for maternal age, parity, pre-pregnancy BMI, folic acid use, smoking, UTI, education, previous miscarriages/stillbirths, maternal mother tongue, year of delivery and vaginal catarrh.

^i^Adjusted for maternal age, parity, pre-pregnancy BMI, folic acid use, smoking, UTI, education, previous miscarriages/stillbirths, maternal mother tongue and year of delivery.

^j^Adjusted for maternal age, parity, pre-pregnancy BMI, folic acid use, smoking, UTI, education, previous miscarriages/stillbirths, length of gestation, maternal mother tongue, year of delivery and vaginal infections.

In stratified analyses according to use of antibiotics against UTI (Table 4), adjusted ORs did not indicate any increased risk of the investigated pregnancy outcomes for the two groups of women: 1) women who had been exposed to cranberry, UTI and antibiotics and 2) women who had been exposed to cranberry and UTI, but not to antibiotics. Crude results detected an increased risk for SGA-infants among the women who had been exposed to cranberry, UTI without use of antibiotics. However, further sub-analyses with adjustment for potential confounding factors were performed with women exposed to UTI without any treatment and women exposed to UTI, antibiotics and no cranberry, revealing no increased risk for having a SGA-infant (adjusted OR 1.0, 95% CI 0.9–1.2 and adjusted OR 1.0, 95% CI 0.9–1.2, respectively).

**Table 4:** Pregnancy outcome according to cranberry use, UTI and use of antibiotics, n = 68,522*

		**No exposure to cranberry and UTI (n = 60,846)**			**Exposed to cranberry, UTI and antibiotics against UTI during pregnancy (n = 254)**			**Exposed to cranberry, UTI and no antibiotics against UTI during pregnancy (n = 300)**		
**Outcome**	**Total (%)**	**No. (%)**	**Crude OR**	**Adjusted OR**	**No. (%)**	**Crude OR (95% CI)**	**Adjusted OR (95% CI)**	**No. (%)**	**Crude OR (95% CI)**	**Adjusted OR (95% CI)**
**Stillbirth/neonatal death**^**a**^	323 (0.5)	293 (0.5)	Ref.	Ref.	1 (0.4)	0.8 (0.1–5.8)	0.8 (0.1–6.2)^g^	3 (1.0)	2.1 (0.7–6.5)	2.2 (0.7–6.6)^g^
**Preterm birth**^**b**^	3,535 (5.2)	3,124 (5.2)	Ref.	Ref.	15 (6.0)	1.2 (0.7–2.0)	1.1 (0.6–1.9)^h^	19 (6.4)	1.2 (0.8–2.0)	1.2 (0.8–2.0)^h^
**Low birth weight**^**c**^	2,182 (3.2)	1,934 (3.2)	Ref.	Ref.	3 (1.2)	0.4 (0.1–1.1)	0.5 (0.2–1.5)^g^	7 (2.4)	0.7 (0.3–1.5)	0.5 (0.2–1.0)^g^
**Small for gestational age**^**d**^	4,281 (6.2)	3,784 (6.2)	Ref.	Ref.	11 (4.3)	0.7 (0.4–1.3)	0.6 (0.3–1.1)^i^	30 (10.0)	**1.7 (1.1–2.4)**	1.4 (0.9–2.1)^i^
**Low Apgar score**^**e**^	898 (1.3)	792 (1.3)	Ref.	Ref.	5 (2.0)	1.5 (0.6–3.7)	1.5 (0.6–3.8)^g^	7 (2.3)	1.8 (0.9–3.8)	1.5 (0.7–3.4)^g^
**Neonatal infections**^**f**^	870 (1.3)	770 (1.3)	Ref.	Ref.	3 (1.2)	0.9 (0.3–2.9)	0.8 (0.3–2.7)^j^	7 (2.3)	1.9 (0.9–4.0)	1.8 (0.9–3.9)^j^

*Abbreviations*: *CI* confidence interval, *OR* odds ratio, *Ref.* reference category.

*Odds ratios (ORs) resulting from Generalized estimating equations analyses are presented in the table. Significant findings are in bold.

The outcomes were not mutually exclusive.

^a^Includes infants who were stillborn with a gestational age of ≥22 weeks or birth weight of ≥425 g, or who died during the first 28 days of life.

^b^Includes infants born at a gestational age of <37 weeks, which WHO defines as preterm.

^c^Includes infants with a birth weight of <2500 g, which WHO defines as low birth weight.

^d^Includes infants with a birth weight below the 10^th^ percentile at the attained gestational age.

^e^Includes infants who had an Apgar score of < 7 at 5 min after birth.

^f^Includes infants who were diagnosed with ICD-10 P35-39.

^g^Adjusted for maternal age, parity, pre-pregnancy BMI, folic acid use, smoking, education, previous miscarriages/stillbirths, length of gestation, maternal mother tongue and year of delivery.

^h^Adjusted for maternal age, parity, pre-pregnancy BMI, folic acid use, smoking, education, previous miscarriages/stillbirths, maternal mother tongue, year of delivery and vaginal infections.

^i^Adjusted for maternal age, parity, pre-pregnancy BMI, folic acid use, smoking, education, previous miscarriages/stillbirths, maternal mother tongue and year of delivery.

^j^Adjusted for maternal age, parity, pre-pregnancy BMI, folic acid use, smoking, education, previous miscarriages/stillbirths, length of gestation, maternal mother tongue, year of delivery, vaginal infections.

## Discussion

Our findings are reassuring, showing no increased risk of congenital malformations, stillbirth/neonatal death, preterm delivery, low birth weight, small for gestational age, low Apgar score and neonatal infections. To the best of our knowledge, this is the first study to investigate the risk of malformations after use of cranberry during pregnancy. These results are in agreement with the safety findings of a prior pilot study of the efficacy for the prevention of ASB in pregnancy showing no differences between the cranberry groups and the control group with regard to obstetric or neonatal pregnancy outcomes [28]. However, we did find an increased risk of vaginal bleeding occurring after pregnancy week 17 among the women who used cranberry during late pregnancy. This association was no longer significant after adjustment was made.

Previous studies have indicated an interaction between cranberry and warfarin [20,32,33,44]. The mechanism of the interaction remains elusive, and different mechanisms are mentioned in the literature [32,33,44]. Cranberry contains significant amounts of salicylic acid, and might increase the risk of bleeding through its capacity to inhibit platelet aggregation [20,32,45]. On the contrary, others state that salicylic acid does not share the antiplatelet effect of acetylsalicylic acid [44,46]. Another biologically plausible mechanism of action has been proposed by Abdul et al. who observed a non-significant trend towards decreased activity of clotting factors when cranberry was co-administered with warfarin [33]. However, the authors did not find any significant independent effect on the clotting system when cranberry was administered alone during the pre-treatment period. Consequently, a clear explanation of the findings with regards to bleeding in this current study is difficult to obtain, but it cannot be completely ruled out that use of cranberry during pregnancy might increase the risk of maternal vaginal bleeding. In the current study, the doses and form of administration were unknown. Vaginal bleeding was self-reported and included spotting in addition to more severe bleeding incidents. We did not find a statistically significant association between use of cranberry during early pregnancy and maternal vaginal bleeding, or between use of cranberry during late pregnancy and more severe bleeding outcomes such as vaginal bleeding more than spotting after pregnancy week 17 and hospitalization due to vaginal bleeding after pregnancy week 12. These findings are reassuring. Nevertheless, a non-significant trend was seen between use of cranberry during late pregnancy and vaginal bleeding more than spotting after pregnancy week 17. Consequently, maternal vaginal bleeding is something that should be explored in later studies with respect to administration form and dosage.

There was no increased risk of malformations in general, major malformations or cardiac malformations among the women who had used cranberry during early pregnancy. However, there was an increase in the proportion of malformations in general in the group of women who had ingested cranberry during the first trimester. Reassuringly, adjusted analyses showed no significantly increased risk for either malformations in general or for major malformations. Furthermore, the malformations varied in nature and did not reveal any clear pattern.

Of note, the women who used cranberry were more likely to be primiparous, to have a college education, and less likely to smoke daily. These findings are in accordance with studies characterizing the users of complementary and alternative medicine in general [3,6,47,48]. Additionally, the association between use of cranberry and use of folic acid is in agreement with prior results showing an association between the use of herbs during pregnancy and use of multivitamins [49]. More women who used cranberry gave birth during the period 2003 to 2006 compared to the period 1999 to 2002. This observation mirrors the frequent use of CAM seen during the recent years in the general population in many Western countries [50-52].

The proportion of women who report treatment with antibiotics in relation to UTI (55.9%) is worryingly low. Pregnant women are previously found to overestimate the risk of medicines [53,54], which may explain this finding. Furthermore, a considerable proportion of the women who used cranberry reported to have experienced a UTI during pregnancy, and many of these women did not report use of antibiotics against this condition. Of note, this group had ORs above 1 for five out of six outcomes shown in Table 4, which may be explained by improper treatment of UTI and the possible harmful effect of UTI on several pregnancy outcomes as suggested by previous studies. However, the adjusted ORs did not show any significant results. Crude ORs showed an increased risk for having a SGA-infant among the women who had been exposed to cranberry, UTI and no antibiotics. However, adjusted analyses and further sub-analyses as previously described did not find any association. This is in accordance with Schieve et al., who found a significant increased risk for having a SGA-infant among women exposed to UTI during pregnancy, which was non-significant after adjustment [12]. Nevertheless, cranberry should not be used to treat UTIs because there is no scientific evidence to support this use [25]. Bacteriuria has been associated with adverse pregnancy outcomes such as low birth weight and preterm delivery [11-14], and untreated ASB is seen to progress to pyelonephritis in 20–40% of pregnant women [9,55]. Pyelonephritis is a serious medical complication of pregnancy and a common reason for hospitalization during pregnancy [9,56]. However, antibiotic treatment of ASB is seen to reduce the incidence of pyelonephritis and the incidence of low birth weight infants [11,17], implying the importance of proper treatment of this condition during pregnancy.

This study has several strengths and limitations. The main strength of the study is the large sample size of the cohort. The risk of recall bias was reduced as a consequence of the prospective nature of data collection in the first two questionnaires completed by the women during pregnancy. However, as the third questionnaire was completed 6 months after giving birth, there might be some recall bias. This could only occur among the 69 women who reported cranberry use between completion of the second questionnaire and delivery in the third questionnaire. This represents 7.5% of the women who used cranberry in pregnancy. Additionally, the vast variety of information on socio-demographic data and pregnancy-related variables, derived from both the detailed MoBa-questionnaires and MBRN, enabled controlling for important potentially confounding factors while performing multivariate analyses on the association between use of cranberry during pregnancy and the selected adverse pregnancy outcomes including malformations. MBRN has been shown to have satisfactory accuracy, as reported by different validation studies [57,58]. Still, it cannot be ruled out that there is a possibility of under-reporting of minor malformations is likely to occur, especially among early stillbirths.

A limitation of MoBa is the low response rate, which may give rise to selection bias. Though minor differences in prevalence estimates are seen between the participants in MoBa and the general population, risk estimates have been shown to be valid for the MoBa data set [59]. Another limitation of the study is that MoBa is based upon self-reporting. Therefore, information on use of cranberry and the diagnosis UTI may not be complete as the medical diagnosis of ASB or symptomatic bacteriuria have not been confirmed. Additionally, the doses and administration forms were not available. The total daily dose, duration of treatment, frequency of treatment and adjunct herbals ingested along with cranberry are therefore uncertain. Furthermore, we did not include dietary intake of cranberry products (i.e. juice, berries) in our study, although this could have been a potential important confounder to adjust for. Lastly, though it is the largest study identified to investigate the safety of cranberry, there were few cases with malformations.

## Conclusions

In conclusion, because of the widespread use of cranberry during pregnancy, the results of this study are of clinical relevance. Even though there is no clear scientific evidence to support the use of cranberry either for the prevention of, or in the treatment of UTIs, pregnant women will probably continue to use this herb because of the long-term history of its use. The findings of this study, revealing no increased risk of malformations or any of the following pregnancy outcomes: stillbirth/neonatal death, preterm delivery, low birth weight, small for gestational age, low Apgar score and neonatal infections, are therefore reassuring. However, maternal vaginal bleeding should be investigated further before any firm conclusions can be drawn. Although pregnant women should be strongly encouraged to use antibiotics against any detected urinary tract infection, cranberry does not appear to be a harmful adjunctive self-therapy with regards to our data and the pregnancy outcomes studied.

## Abbreviations

ASB: Asymptomatic bacteriuria; CI: Confidence interval; MBRN: The medical birth registry of Norway; MoBa: The Norwegian mother and child cohort study; OR: Odds ratio; SD: Standard deviation; SGA: Small for gestational age; UTI: Urinary tract infection.

## Competing interests

The authors declare that they have no competing interests.

## Authors’ contributions

KH performed and interpreted the statistical analyses and drafted the manuscript. HN participated in the design of the study, contributed assistance regarding performing and interpreting the statistical analyses, and revised the manuscript critically. LH participated in the design of the study, helped to interpret the results from the statistical analyses and revised the manuscript critically. All authors participated in the review of all herbal products for cranberry as an ingredient and read and approved the final manuscript.

## Authors’ information

• KH, PhD-student, MScPharm, did her master in pharmacy, University of Bergen, Norway on attitudes and use of herbal medicines during pregnancy. She is now a PhD-student at the Department of Global Public Health and Primary Care, Faculty of Medicine, University of Bergen, Norway.

• HN, Dr.Philos., McScPharm, is a professor at the School of Pharmacy, University of Oslo, Norway and researcher at the Division of Mental Health, Norwegian Institute of Public Health, Oslo, Norway. The focus of her research is medication use and safety during pregnancy and breastfeeding.

• LH, PhD, MScPharm, is an associate professor at the Department of Global Public Health and Primary Care, Faculty of Medicine, University of Bergen, Norway. The focus of her research is herbal medicine use and safety during pregnancy.

## Pre-publication history

The pre-publication history for this paper can be accessed here:

http://www.biomedcentral.com/1472-6882/13/345/prepub
